# Supplementary figures and images for: Bioinformatic characterization of angiotensin-converting enzyme 2, the entry receptor for SARS-CoV-2
Source: PLoS One. 2020 Oct 28;15(10):e0240647. doi: 10.1371/journal.pone.0240647 (PMC7592753; doi:10.1371/journal.pone.0240647)

A

homo\_sapiens  
ACE2-201 ENST00000252519

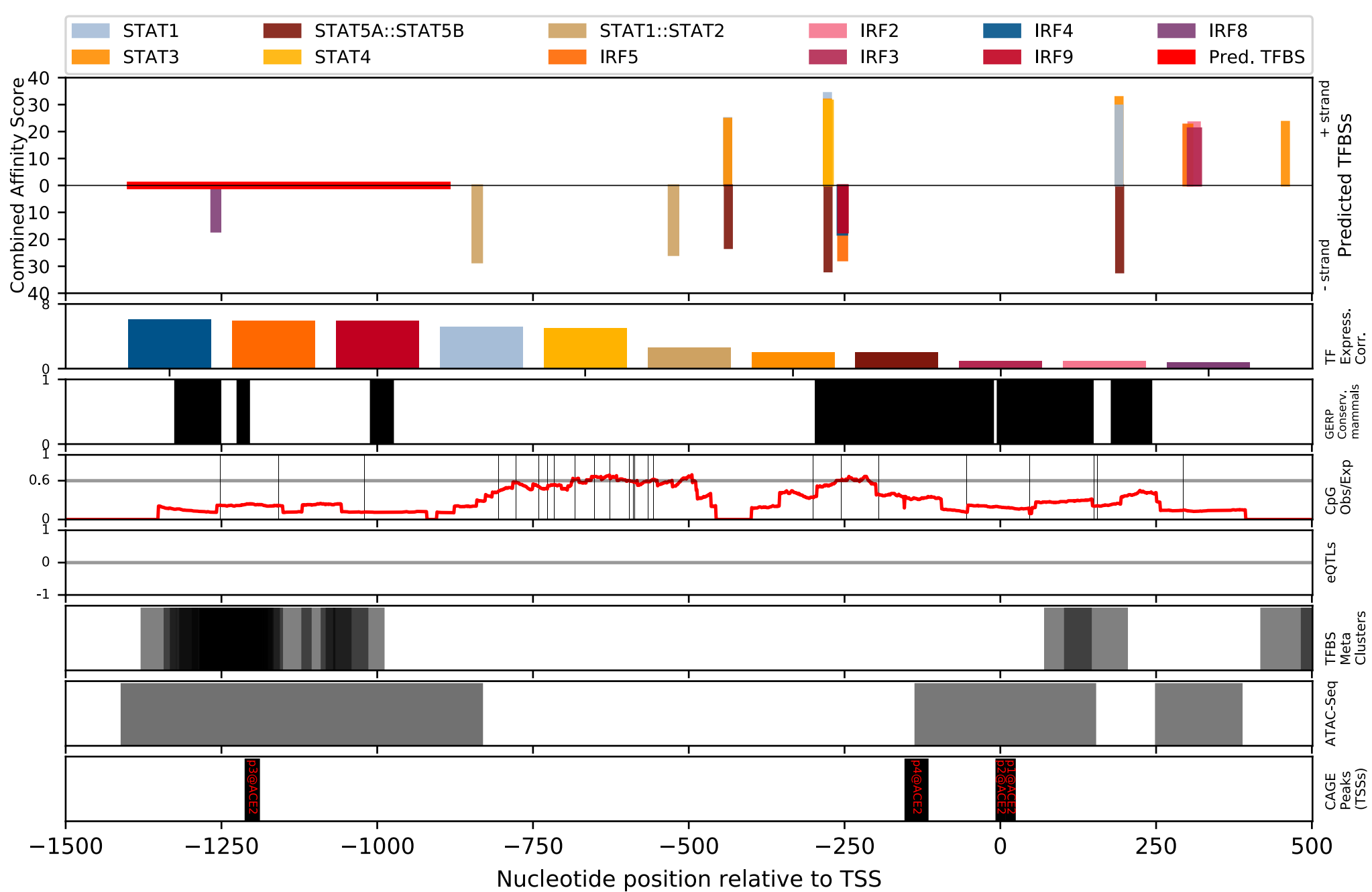

B

homo\_sapiens  
ACE2-202 ENST00000427411

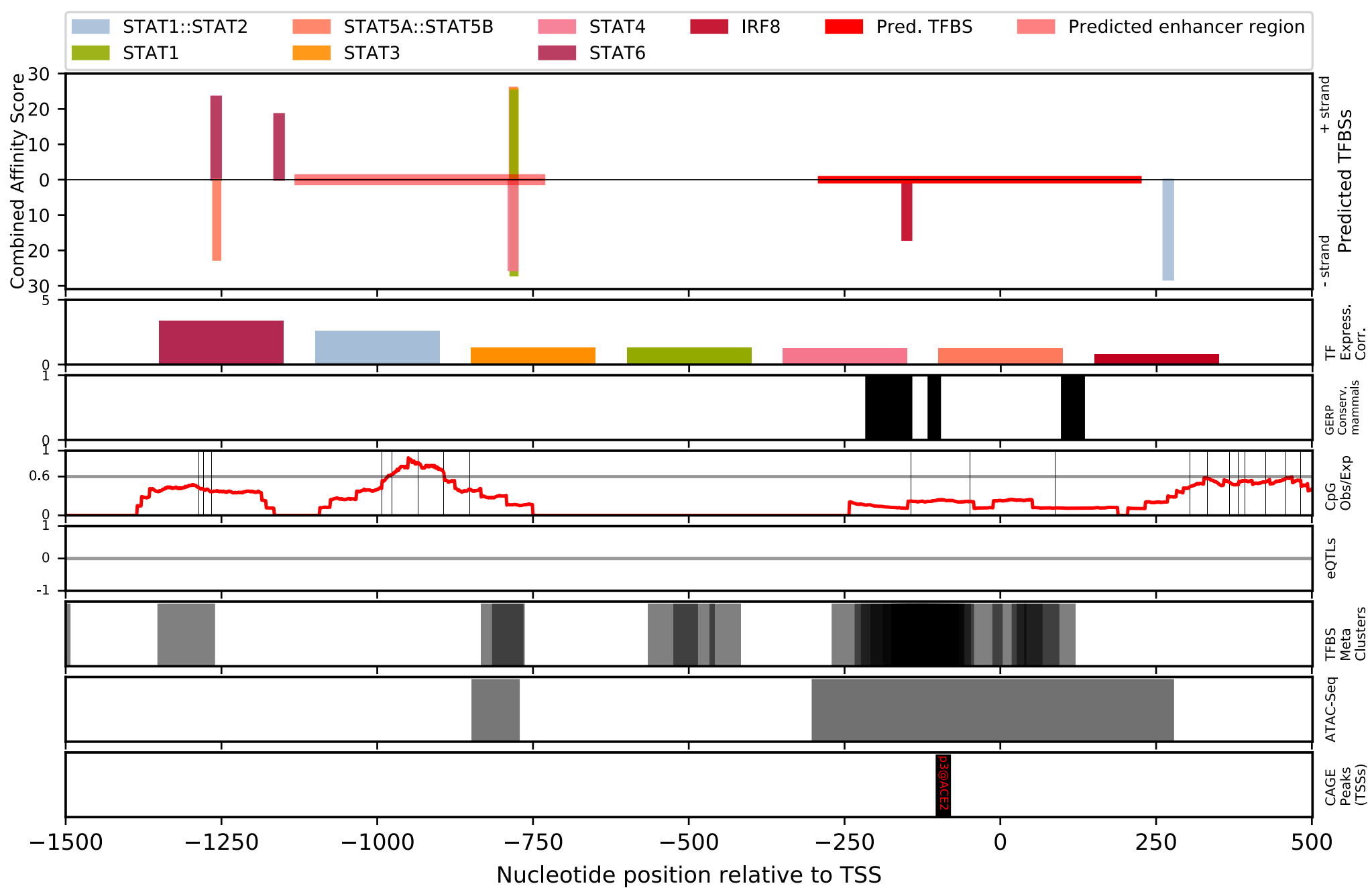

Supplement: S1 Fig — (PDF) [file pone.0240647.s001.pdf]
