# Supplementary material for: Bioinformatic characterization of angiotensin-converting enzyme 2, the entry receptor for SARS-CoV-2
Source: PLoS One. 2020 Oct 28;15(10):e0240647. doi: 10.1371/journal.pone.0240647 (PMC7592753; doi:10.1371/journal.pone.0240647)
Supplement: Attachment — Submitted filename: Response to reviewers.docx [file pone.0240647.s010.docx]

**Reviewer #1:**

1. Figure 3 & 4: is not clear, the image quality is not good and different cell types is not readable.

OUR RESPONSE: We have now prepared these images using the Preflight Analysis and Conversion Engine (PACE) digital diagnostic tool, ensuring figure quality according to the requirements of PLOS ONE.

2. What is the reason behind using both human and mouse tissues for the study? was there any difference between the ACE2 expression in mouse and human in different organs? Please comment.

OUR RESPONSE: Experiments on tissues from mouse and human represent the greatest proportion of single-cell RNA-Seq datasets available, a total of 937 experiments out of 1,038 (<https://www.nxn.se/single-cell-studies>). Of these, experiments with data from mouse make up the larger proportion, a total of 563 experiments. The reason of selecting these particular mouse datasets for the single cell RNA-Seq analysis was the better quality of the dataset compared to human ones. Several scRNA-Seq datasets representing lung/airway tissue were reviewed before settling on the datasets which are included in this manuscript. In all of these cases, the number of lung cells which express ACE2 were exceptionally low, often representing just 1–2% of the total cells in the analysis. One of the primary reasons we chose the upper airway/trachea dataset presented in our manuscript was that it had the highest proportion of ACE2-expressing cells. We hoped that this would give a better chance at clearly identifying those cell subtypes which ACE2 is characteristic for.

Direct comparison of mouse and human tissues can be difficult due to lack of age/sex data and different sampling techniques. However, comprehensive sampling of both species has been performed by the FANTOM5 project. Our comparisons of ACE2 expression in mouse and human tissues (FANTOM5 dataset) have shown that generally ACE2 mRNA is expressed similarly across tissues. Differences are seen in sex related tissues; testis (higher expression in human) and uterus and vagina (higher expression in mouse), but are similar in lung (low expression) and gastrointestinal tissues (high expression). This is in accordance with a meta-analysis specifically addressing the topic of concordance of human-mouse ortholog expression, which found that at the global level gene expression in human and mouse is conserved (Dowell RD. The similarity of gene expression between human and mouse tissues. Genome Biol. 2011;12(1) 101).

3. The results in the current paper with respect to ACE2 mRNA expression was contrasting with Lukassen and colleague, as they claimed high expression of ACE2 in human respiratory tract, which is the reason behind COVID19 patients experiencing pneumonia. Please explain.

OUR RESPONSE: We have cited Lukassen and colleagues´ study in the discussion section. We think that there is no major discrepancy between our and Lukassen et al. results, but the cells included in the datasets have been defined in a slightly different manner. Overall, the ACE2 expression is low in the respiratory tract. This is also clearly stated by Lukassen et al: “All major cell types known to occur in the lung were identified (Fig 1B and C). Independent of the cell types present in the lung, the median *ACE2* levels were below five counts per million (CPM) (Fig 1D), which given a typical mRNA content of 500,000 mRNA molecules per cell indicate that only about half of all cells were statistically expected to contain even a single *ACE2* transcript.” We have further stated in the discussion section that the dataset used in our study did not identify secretory3 cells which showed the highest expression in the Lukassen et al. study. (Page 21, lines 36-38)

4. Page 21, line 56-71, the authors conclude the high expression of ACE2 in Club, goblet and ciliated cells of trachea, which can cause respiratory distress, but the overall expression of ACE2 in figure 1 is highest in small intestine. What could be the reason behind the COVID19 patients not experiencing the gastrointestinal symptom as seen in SARs-CoV-1. The Club, goblet and ciliated cells are also expressed in the intestine. What could be the reason behind COVID19 infection severely affecting lungs?

OUR RESPONSE: The referee raised an interesting question. We are not aware of the presence of Club cells and ciliated cells in the intestine. It is well known that absorptive enterocytes have apical microvilli but no cilia. Both respiratory and gastrointestinal tracts have goblet cells whose main function is the same, i.e. production of mucus. Based on our analysis, 13.84% of tracheal goblet cells express ACE2 (Page 11, line 237), whereas only 1.35% of the intestinal goblet cells were ACE2-positive (Page 12, line 258). We have no clear opinion what could be the reason that COVID-19 patients have more rarely gastrointestinal symptoms compared to SARS-CoV-1-infected patients. We have stated this in the Discussion section (Page 23, lines 77-79).

The next question about the reason behind COVID-19 infection severely affecting lung is one of the key questions raised by researchers during the pandemic. There may be several possible mechanisms, of which clotting of microvessels may be the most relevant at this moment. It has been convincingly shown that COVID-19 severely affects microcapillaries also in the lung. The first autopsy series reported following COVID-19-related deaths described extensive diffuse alveolar damage and thrombi within small peripheral vessels in the lungs. To emphasize this feature of COVID-19 we have added the following sentences: “Our GO analysis provided evidence that ACE2 is involved in the KEGG pathway 'complement and coagulation cascades'. Indeed, patients with severe COVID-19 often present with coagulation abnormalities that mimic other known systemic coagulopathies, such as disseminated intravascular coagulation (DIC) or thrombotic microangiopathy, but COVID-19 has its own distinct features (90).” (Page 26, lines 154-158)

5. The authors concluded, no difference in ACE2 expression was observed between the age groups and genders. Please reconcile with COVID19 reports adversely infecting older population and men.

OUR RESPONSE: We provide the following statement in the Introduction: “It is known that both SARS-CoV and SARS-CoV-2 infections lead to worse outcome in the elderly (30,34).” (Page 5, lines 107-108)

We have added and modified the following sentences: “Recent studies have also indicated higher case fatality rates in males than females (35). Therefore, one aim of the present study was to investigate whether age or gender could contribute to the regulation of ACE2 expression.” (Page 5, lines 108-110)

We state in the discussion section: “Since upregulation of ACE2 would reflect an increased number of SARS-coronavirus receptors on cell surfaces, it could possibly help us to understand the mechanisms why certain patients (males more than females, old more than young, smokers more than non-smokers) are more susceptible to the most detrimental effects of the COVID-19 infection. In our study, the signals for ACE2 mRNA in the lung specimens did not vary much in different age groups nor did they show significant differences between males and females, which is in line with previous findings (62). Therefore, different expression levels of lung ACE2 may not explain the variable outcome of the disease concerning age groups and genders. Importantly, our studies on this aspect were performed using whole tissue RNA-Seq values, and at least one other analysis using single-cell RNA-Seq data has identified changes in ACE2 expression associated age, sex, and smoking status for various cell types (70). Specifically, they have found ACE2 expression to increase with age in basal and multiciliated cells, and higher expression for males in airway secretory cells and alveolar AT2 cells. Additionally, a study of ACE2 expression in nasal epithelium (not included in GTEx dataset) showed lowest levels in young children (<10) with increasing values in later age groups (71). It has been recently discussed that different innate and adaptive immune responses related to both age and gender may contribute to variable outcome of severe viral diseases (35). It is clearly one major research area to be followed regarding COVID-19 infection.” (Pages 23-24, lines 82-98)

6. The authors identified the possible transcription factor binding site, which drive ACE2 expression in lung and intestine. The figure 9 showed MGST1, MTUS1, and MGAM could be potential site, which needs to be discussed further. As I see the discussion part is highlighting the findings from other papers.

OUR RESPONSE: We believe that these three genes, which have high co-expression with ACE2 and high expression in the lung and intestine, may be relevant in terms of biological processes which ACE2 is involved in but are not likely relevant in terms of transcriptional regulation, as they are not themselves transcription factors. For example, MTUS1 directly interacts with angiotensin II.

7. Expression of ACE2 in the current study does not match original reports of ACE2 expression (Harmer et al. 2002). Please explain.

OUR RESPONSE: We thank the reviewer for introducing this important paper which is now included as a reference. We feel that there is no major discrepancy between Harmer et al. and our results. Using qPCR they showed the highest expression levels of ACE2 in testis, kidney, cardiovascular tissues, and intestine. Slight differences could be expected due to different techniques and biological specimens. We have added the following sentence: “Quantitative polymerase chain reaction (qPCR) showed the highest expression levels in the human cardiovascular system, testis, kidney and intestine (26)” (Page 4, lines 88-90)

**Reviewer #2:**

In this paper, a secondary data analysis on human and mouse data sets was performed in regard to ACE2 mRNA expression using bioinformatics tools. The authors attempted to determine cell-specific expression of ACE2 mRNA in a number of organs that could likely be viewed as for SARS-Cov-2 virus entry ports. Prediction of transcription factor binding sites, gene ontology and sex-comparative analysis were also performed. The authors hypothesized that using these bioinformatic tools one could predict functions and transcriptional regulation of ACE2 yet not all approaches had been shown to be validated yet (see below).

The introduction clearly delineates the rationale for the studies performed. The Discussion is well structured and gives a logical summary of the presented findings. Less organized is the Results section. The data presented here also reveals some rigor deficiencies which makes the paper less convincing. Also, the approach has to be critically viewed by cautioning that other levels than transcriptional could be operative in regulating expression of ACE2.

In summary, the paper provides some interesting data and conclusions. However, there are deficiencies that need to be addressed.

Specific comments:

• Many studies, and also the Protein Atlas website, a tool the authors used themselves, have reported high ACE2 protein expression in the kidney yet the ACE2 mRNA expression in the kidney was reported here as relatively low. By unsophisticated comparison, there is a discrepancy that might suggest a level of regulation beyond the transcription. Moreover, there have been reports on post-transcriptional regulation of ACE2, e.g. in diabetes. It should be stated that one of the limitations of the approach used is that it is based on mRNA expression and transcriptional regulation only and that other levels of ACE2 regulation could be effective as well.

OUR RESPONSE: We are grateful for this comment. The reviewer is absolutely right that there may be regulation beyond the transcription that can affect the protein expression levels. We added the following information: “One limitation of our study is that it is focused on mRNA expression and transcriptional regulation only. There may exist factors which function at posttranscriptional level. Indeed, Srivastava and colleagues recently demonstrated that SARS-CoV-2 infection induces alterations in the post-transcriptional regulatory networks in human tissues through the function of RNA binding proteins and micro-RNAs (73).” (Page 25, lines 127-131)

• The novel computational tool that has been recently developed by the first author of this article has not been published. Yet the authors use it to predict transcriptional regulation. Has this tool been validated in any way? How this tool works and how was it verified that the tool really works. If the tool has not been published and no data on its validation has been provided, should the reader just take it for granted? Some information has to be given on this or, if already available, the appropriate reference cited.

OUR RESPONSE: We agree that this is a valid question regarding the use of an unpublished tool. We have since submitted the original article detailing the TFBSfootprinter program titled ‘Evolution is in the details: Regulatory differences in modern human and Neanderthal’ to bioRxiv as a pre-print (reference #48)

The TFBSfootprinter tool has been benchmarked using several datasets, including TFBS ChIP-Seq peaks compiled in the GTRD database, as well as the experimentally validated TFBSs of the ORegANNO, Pazar, and ABS datasets. Using these datasets as benchmark, the TFBSfootprinter tool has been compared with traditional position weight matrices, which it outperforms in all cases, as well as the deeplearning-based DeepBind program, which it outperforms in most cases. Identification of transcription factor binding sites is not a new field, and the TFBSfootprinter program uses additional transcription-relevant data to make modest improvements in the accuracy of prediction in addition to the well-documented traditional PWMs. In addition, results from our tool have been included in several previously published articles (Barker et al. Role of carbonic anhydrases in skin wound healing. Exp Mol Med 2017;49:e334; Karjalainen et al. Carbonic anhydrase related protein expression in astrocytomas and oligodendroglial tumors. BMC Cancer 2018;18:584).

• Given that many of the findings have not yet been verified in vivo or in vitro, it would be appropriate to mention that the validity for the majority of the findings presented here would need to be verified by future research.

OUR RESPONSE: We thank the reviewer for this comment. Indeed, many experimental studies will be required to verify these findings. Therefore, we added the following sentence: “Many findings reported here have not yet been verified *in vivo* or *in vitro*. Therefore, the validity of the bioinformatics results need to be verified by future experimental research.” (Page 27, lines 173-175)

• In this paper, a secondary data analysis on human and mouse dataset studies was performed. It takes a bit effort for the reader to find out what was studied. Given that various sources that were used for this, it would be useful to give some overview in form of a table or so: the source of data set, species, what was studied.

OUR RESPONSE: Thank you for your insight regarding the clarity of data presentation. We have created a table which outlines these data sources, which has been included as Supplementary Table 1. We have added the following text: “(description of all public datasets used presented as Supplementary Table 1)”. (Page 6, line 120).

• There is insufficient information how the authors performed evaluation of the images that can be accessed from the Protein Atlas. How many images assessed, what magnification etc.

OUR RESPONSE: We have added the numbers of images available in the Protein Atlas (Page 7, lines 142-144). We have also added bars indicating 100 µm to each image.

• Lanes 119 and 125 - Information is missing on what is GTEx database and where can be found.

OUR RESPONSE: A statement on the GTEX dataset has been added: “which is an ongoing large-scale project to identify human variation, regulation, and gene expression” (Page 6, lines 124-125)

• Is the expression of ACE2 in the kidney moderate (Abstract, Figure 2) of one of the highest (Discussion, Conclusions)?

OUR RESPONSE: We have re-evaluated the immunostaining results of kidney and compared to other tissues, and indeed, it can be stated to be in the same (strong) intensity category with duodenum and testis. Therefore, we have replaced the word “prominent” with “strong” (Page 10, line 208 and Page 11, line 219). We also clarified these results in the abstract section: “The small intestine expressed higher levels of ACE2 mRNA than any other organ. By immunohistochemistry, duodenum, kidney and testis showed strong signals, whereas the signal was weak in the respiratory tract” (Page 2, lines 28-31).

**Reviewer #3:**

In their manuscript, Barker and Parkkila investigate the expression and transcriptional regulation of ACE2, the receptor of SARS-CoV-2, through the analysis of existing datasets. Specifically, they determine the tissue and cell type specificity, investigate the covariates age and gender, and identify putative transcriptional regulators of ACE2 as well as genes with highly correlated expression patterns.

The manuscript is generally well written and nicely merges different layers of information on the expression and potential role of ACE2. The description of statistical tests is thorough, and appropriate tests are used for the gender and correlation analyses. For the analysis of age dependency, the one-way ANOVA implemented in scipy.stats is not an optimal choice as the ranking information of the age bins is lost. Ideally, the authors should try to work with the raw values if available and perform a regression fit here. Looking at the box plots, this should work reasonably well even with simple linear regression for most tissues, with the exception of kidney. Parts of the manuscript would benefit from a more detailed description of the approach taken, and some results contradict prior literature which would need to be addressed. Furthermore, as ACE2 is discussed specifically in its function as SARS-CoV-2 receptor, the manuscript could benefit from further analyses geared more towards ACE2 regulation in the context of an infection. I would not consider the latter point to be strictly necessary, provided the authors clearly state that their analysis is a representation of the physiological expression pattern and may not fully hold during a SARS-CoV-2 infection, but if it can be done quickly it would be worth considering.

Specific comments:

• It is sometimes unclear which dataset an analysis was performed on. In general, a structured list of datasets including links to the resources in the Methods section or as a supplementary table would be a great help.

OUR RESPONSE: Thank you for your insight regarding the clarity of data presentation. We have created a table which outlines these data sources, which has been included as Supplementary Table 1. We have added the following text: “(description of all public datasets used presented as Supplementary Table 1)”. (Page 6, line 120).

• For the TFBS analysis, I’m not sure what the tissues in parentheses (e.g. lines 325/326) indicate. Was a correlation observed in these tissues (if yes, was the dataset bulk or single-cell)?

OUR RESPONSE: Indeed, the tissues in parenthesis after gene names are those where ACE2 and that gene are highly coexpressed. This section has been clarified with: “...as determined using RNA-Seq gene expression values from the GTEX dataset...”. (Page 15, lines 318-319).

• It remains somewhat unclear how TFBS footprinting works. Based on the GitHub repository it mostly seems to aggregate PWM scores with experimental measures for TF occupancy. Given that there is no paper or preprint yet, the results presented in this manuscript should be explained in more detail (e.g., what data is the ATAC-Seq track based on? Is it lung or some other organ/aggregate database?). In addition, some corroborating evidence, or a rationale for why this is not needed, i.e. the data shown can directly be obtained from a database (which?), should be provided.

OUR RESPONSE: We have since submitted the original article detailing the TFBSfootprinter program titled ‘Evolution is in the details: Regulatory differences in modern human and Neanderthal’ to bioRxiv as a pre-print (reference #48)

The TFBSfootprinter tool was used as it has proven to be more accurate than existing methods. We have benchmarked it using both a large set of ChIP-Seq peaks compiled in the GTRD dataset as well as experimentally validated functional TFBSs compiled in the Pazar/ORegAnno/ABS datasets. We have updated the methods with more detail on the usage of ATAC-Seq data: “...to enhance prediction of putative TFBSs, including: all cell types aggregated and merged human ATAC-Seq data from ENCODE...” (Page 7, lines 154-155).

• ACE2 has been described as an interferon responsive gene. This could have implications when transferring the results presented here to the situation in COVID-19, as no major interferon signaling would be expected in healthy tissues. This could also help explain the disparity between the severity of COVID-19 and the lack of ACE2 expression in the lung beyond the initial infection. Several STAT/IRF TFs appear to have a significant hit according to the PWM scoring (and in the case of STAT1 for combined affinity). Since this interferon-responsiveness has now been found by several studies, it should be discussed, or directly addressed through inclusion of an infected dataset. Ideally that would be one of the COVID-19 datasets slowly becoming available, but if the question is just about interferon response, other infections such as influenza should work as well.

OUR RESPONSE:

This is a fascinating and important connection that had escaped our attention. We thank the reviewer for this comment and suggestion and have subsequently made an analysis (Supplementary figure 1) of the two tissue-specific promoters with only the interferon regulatory TF genes. Accordingly, statements have been added to the Methods (Page 8, lines 164-165), Results (Page 15, lines 324-329 and Page 16, lines 353-358), and Discussion (Page 25, lines 117-125).

**Abstract statement:**

These include several interferon-stimulated response elements sites for STAT1, IRF8, and IRF9. (Page 2, lines 38-39)

**Methods statement:**

These two transcripts were targeted for transcription factor binding site (TFBS) analysis; first with a scan for all 575 Jaspar TFs and input parameters of 1,000 base pairs (bp) upstream and 200 bp downstream, (relative to the TSS); secondly with a limited set of 15 interferon-stimulated TF genes and a broader area of 1,500 bp upstream and 500 bp downstream. (Page 8, lines 164-165)

**Results statement (I):**

An expanded analysis for binding sites of interferon-stimulation mediating TF genes in the region 1,500 bp upstream to 500 bp downstream of the ACE2 intestinal transcript’s TSS revealed putative binding sites for STAT1 (3x), STAT3 (4x), STAT4, IRF2, IRF3, IRF4 (2x), IRF5 (2x), IRF8 (3x), and IRF9 (Supplementary Figure 1). Each of these predictions satisfied a PWM p-value threshold of <0.0001, while only the STAT1, IRF9, and IRF8 sites also satisfied a combined affinity score p-value of <0.05. (Page 15, lines 324-329)

**Results statement (II):**

An expanded analysis for binding sites of interferon-stimulation mediating TF genes in the region 1,500 bp upstream to 500 bp downstream of the lung transcript’s TSS revealed putative binding sites for STAT1 (2x), STAT3, STAT4, STAT6 (2x), and IRF8 (Supplementary Figure 1). Each of these predictions satisfied a PWM p-value threshold of <0.0001, while only the IRF8 site also satisfied a combined affinity score p-value of <0.05. (Page 16, lines 353-358)

**Discussion statement:**

Recent work has shown that ACE2 expression is stimulated by interferon alpha (IFN-α) in vitro and computationally identified evidence for STAT1, STAT3, IRF8, and IRF1 TFBSs in the ACE2 promoter [76]. Another recent study has identified correlations of expression between ACE2 and other interferon stimulated genes [77]. Our analysis produced several putative binding sites for interferon-stimulation mediating TF genes, proximal to the TSSs of the intestine-specific (STAT1, IRF8, and IRF9) and lung-specific transcript (IRF8). The findings of these studies, and our own, potentially reveal a scenario where SARS-CoV-2 infection itself may induce expression of ACE2 and thus provide a self-perpetuating route of increased cellular infection. This could explain how such low overall ACE2 expression in normal lungs translates into a fatal disease state. (Page 25, lines 117-125)

• The findings on the influence of age and gender contradict some existing literature on the subject (Muus et al., bioRxiv 2020; Bunjavanich et al., JAMA 2020; indirectly as they measured activity rather than expression: Soro-Paavonen et al., Journal of Hypertension 2012; Fernández-Atucha et al., Biology of Sex Differences 2017). While I wouldn’t necessarily consider the issue settled yet given the low expression of ACE2 and associated high levels of noise, these disparities should definitely be addressed.

OUR RESPONSE: We thank the reviewer for bringing attention to these other studies, we have added text to the Discussion addressing our use of whole tissue RNA-Seq data, and the findings of these two studies:

“Since upregulation of ACE2 would reflect an increased number of SARS-coronavirus receptors on cell surfaces, it could possibly help us to understand the mechanisms why certain patients (males more than females, old more than young, smokers more than non-smokers) are more susceptible to the most detrimental effects of the COVID-19 infection. In our study, the signals for ACE2 mRNA in the lung specimens did not vary much in different age groups nor did they show significant differences between males and females, which is in line with previous findings (62). Therefore, different expression levels of lung ACE2 may not explain the variable outcome of the disease concerning age groups and genders. Importantly, our studies on this aspect were performed using whole tissue RNA-Seq values, and at least one other analysis using single-cell RNA-Seq data has identified changes in ACE2 expression associated age, sex, and smoking status for various cell types (70). Specifically, they have found ACE2 expression to increase with age in basal and multiciliated cells, and higher expression for males in airway secretory cells and alveolar AT2 cells. Additionally, a study of ACE2 expression in nasal epithelium (not included in GTEx dataset) showed lowest levels in young children (<10) with increasing values in later age groups (71). It has been recently discussed that different innate and adaptive immune responses related to both age and gender may contribute to variable outcome of severe viral diseases (35). It is clearly one major research area to be followed regarding COVID-19 infection.” (Pages 23-24, lines 82-98)

• As ACE2 is rather strongly expressed in the vasculature, I would have been surprised to not see angiogenesis pop up as enriched pathway. While I wouldn’t discount the possibility that ACE2 does indeed play a role in this process, it will be hard to reliably make this point based on the datasets included in this manuscript. While the authors could flesh out their analysis with angiogenesis datasets, ideally showing a differential and stage-specific expression of ACE2 in the process on the RNA and/or protein level. There is a paper describing an ACE2 knockout mouse (Crackower et al., Nature 2002), but the phenotype they describe seems to hint more towards the “canonical” functions of ACE2.

OUR RESPONSE: We thank the reviewer for raising this point, and based on an expanded review of the literature have included statements supporting the GO analysis: "Angiotensin-(1-7) is a direct product of ACE2, and through binding with the Mas receptor has been shown to advance angiogenesis in injured cardiac tissue (myocardial infarction), by increasing expression of VEGF-D and MMP-9 [83], and in stroke [84]. Other studies have suggested that ACE2, either by reducing angiotensin II or through activities of the ACE2/angiotensin-(1-7)/MasR axis, may be negatively associated with angiogenesis in various cancers [78, 85-87]. It also appears to play a role in angiogenesis in uterus during pregnancy [88].” (Pages 26, lines 143-148).

• Fernández-Atucha et al. describe an age-dependent effect on ACE2 activity in females only (by linear regression). It might be worth looking at the interaction term rather than performing a one-way ANOVA here.

OUR RESPONSE: We thank the reviewer for pointing out this additional study addressing the female-specific age-related increase of ACE2 in blood serum. Due to privacy considerations the GTEx demographics data on patient age is not continuous and is only available as age ranges (by 10-year groupings). As a result, we cannot perform good linear regression on this data. However, we have subsequently performed additional separate pairwise comparisons of the same two age groups (<50, >=50) for each sex.

Added text: “Additionally, with the same age groups, comparisons were made for both males and females (Supplementary Table 3). In males, ACE2 expression was lower in the ≥50 age group for tibial nerve (p=1.33 x 10-7), subcutaneous adipose tissue (p=1.23 x 10-4), minor salivary gland (p=4.03 x 10-4), transverse colon (p=4.67 x 10-3), sigmoid colon (p=0.017), testis (p=0.025), visceral adipose tissue of omentum (p=0.026), sun exposed skin (p=0.027), whole blood (p=0.032), and bladder (p=0.042); while increased in coronary artery (p=0.015). In females, ACE2 expression was lower in the ≥50 age group in whole blood (p=0.005) and sun exposed skin (p=0.049); and higher in esophagus (p=0.007) and terminal ileum (p=0.022).” (Page 13, lines 280-288).

Minor comments:

The citation of the primary publication of the human protein atlas appears to be missing (see <https://www.proteinatlas.org/about/licence>).

OUR RESPONSE: We added a reference Uhlen et al. 2015 (Page 7, line 142)
