# Supplementary material for: Bioinformatic characterization of angiotensin-converting enzyme 2, the entry receptor for SARS-CoV-2
Source: PLoS One. 2020 Oct 28;15(10):e0240647. doi: 10.1371/journal.pone.0240647 (PMC7592753; doi:10.1371/journal.pone.0240647)
Supplement: Attachment — Submitted filename: Response to reviewer_2.docx [file pone.0240647.s011.docx]

**Reviewer #3:**

Reviewer #3: The authors have completely and thoroughly addressed my concerns, however, I cannot recommend acceptance of the article yet. I am deeply sorry for raising another issue that wasn't included in the first round of revisions, but days after the decision on the previous version of the manuscript, three preprints (<https://www.biorxiv.org/content/10.1101/2020.07.24.219139v1>, <https://www.biorxiv.org/content/10.1101/2020.07.19.210955v1>, <https://www.biorxiv.org/content/10.1101/2020.07.31.230870v1>) were uploaded to bioRxiv that shed more light on the interferon-dependent regulation of ACE2, independently identifying a previously unknown, likely non-functional isoform of ACE2 that is strongly interferon-responsive. This isoform stems from an alternative promoter, leading to a 5' truncation that is not picked up by 3' (single-cell) RNA-Seq. As this is highly relevant to the question of regulation of ACE2 during COVID-19, I would strongly urge the authors to add an analysis of this alternative promoter. Furthermore, it might be worth looking at GTEx exon-level data at least for tissues where some sort of differential expression is observed, or restrict the comparison to the 5' exons to capture just the full-length isoform. If no major differences are observed, this information could be moved to a supplementary figure.

For this reason, I recommend a major revision. Please note that this is not reflective of the general quality of the work: Had this additional information not been made available in the meantime, I would have happily recommended acceptance of the article as-is.

OUR RESPONSE: We thank the reviewer for this valuable comment. We have revised the manuscript according to the new information. We have added the following texts and other materials (lines correspond to the version with ”track changes”:

Abstract, lines 37-38

Methods, lines 165-166

Results, lines 360-375

Discussion, lines 125-162

New Figure 9 has been added

References 56, 57, 58, and 82 have been added

Supplementary Table 5 has been revised

MINOR COMMENT: The quality of some figures is still rather bad. I think I had a similar issue with Editorial Manager in the past, where the uploaded high quality images were compressed too strongly. If that is the case, everything is fine. If the images in the reviewer file are reflective of the actual quality though, I would urge a shift to vector graphics or at least higher resolution images.

OUR RESPONSE: The figure quality has been poor because of the automated compression for the reviewer version of the manuscript. It shouldn´t be an issue for the final version of the paper.

OTHER MINOR CHANGES:

Gene abbreviations are written in *Italic*.

Supplementary Figure 1 has been updated to include STAT dimers which had been accidentally left out in the first analysis.

The number of COVID cases has been updated (lines 56-57).
